# Enhancing the anti-aging potential of the nigrostriatal dopamine system to counteract age-related motor decline

**DOI:** 10.1038/s41392-025-02234-7

**Published:** 2025-05-12

**Authors:** Youngpyo Nam, Sehwan Kim, Jun-Yeong Lee, Jaekwang Kim, Sang Ryong Kim

**Affiliations:** 1 School of Life Science and Biotechnology, BK21 FOUR KNU Creative BioResearch Group, Brain Science and Engineering Institute, Kyungpook National University, Daegu, Republic of Korea; 2 Dementia Research Group, Korea Brain Research Institute, Daegu, Republic of Korea

**Keywords:** Translational research, Preclinical research, Cellular neuroscience

**Dear Editor**,

Aging is linked to changes in brain function that lead to physical decline, including motor deficits and skeletal muscle loss.^1,2^ While maintaining motor activity is crucial for improving the quality of life in the elderly, there is limited research on strategies to prevent motor function decline and preserve skeletal muscle. Additionally, approaches to preserving the function of critical neural systems to mitigate age-related motor decline remain underexplored.

The nigrostriatal dopamine (DA) system, a critical part of the basal ganglia responsible for regulating motor function, is particularly vulnerable to aging, and its dysfunction plays a significant role in motor activity decline.^3,4^ While direct links to neurodegenerative diseases, such as Parkinson’s disease, still require further clarification and research, these reports suggest that age-related changes in motor function and skeletal muscle are influenced by functional disruptions in the nigrostriatal DA system. However, there is no evidence linking the long-term preservation of DA neuron activity in the substantia nigra pars compacta (SNpc) to the prevention of age-related skeletal muscle loss. To address these gaps, we investigated a crucial factor involved in nigral DA neuron maintenance, aiming to preserve motor function in aging.

We found a marked decrease in DA-related proteins, including tyrosine hydroxylase (TH) and phosphorylated TH (p-TH), in the SNpc of aged mice (20 months old) compared with their younger counterparts (3 months old), indicating a decline in the catalytic activity of DA production with aging (Fig. 1a, upper panels). Notably, the levels of sirtuin 3 (SIRT3), a mitochondrial regulator associated with anti-aging effects,^5^ were significantly reduced within DA neurons in the SN of 20-month-old mice (Fig. 1a, upper panels). This decline in SIRT3 was more pronounced in the SN compared to other brain regions such as the hippocampus and cortex (data not shown). Given that SIRT3 loss is associated with impaired mitochondrial function, oxidative stress, and neurodegeneration in nigral DA neurons,^5^ we hypothesized that restoring SIRT3 expression in these neurons could mitigate the age-related decline of the nigrostriatal DA system and preserve motor function in aged mice.

**Fig. 1:**
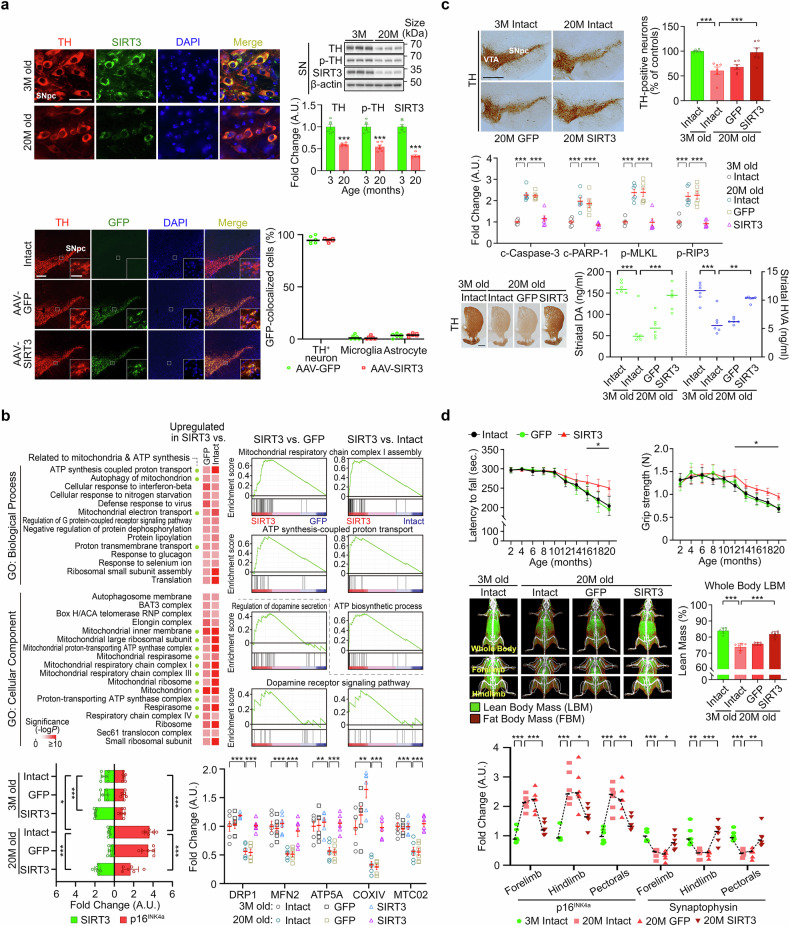
Mitigation of motor decline by preserving nigral DA neurons during aging. **a** Immunofluorescence staining for TH and SIRT3 in the SNpc of young and aged mice (scale bar, 50 μm; left upper panels) and Western blotting of TH, p-TH, and SIRT3 in the SN (right upper panels). A.U., arbitrary unit. Immunofluorescence staining and quantification for cellular colocalization of AAV-GFP and AAV-SIRT3-induced GFP in the SN 4 weeks post-injection (scale bars, 200 μm and 50 μm; lower panels). **b** GO enrichment analysis for BP, CC, and GSEA of ATP production and DA signaling in the SN 4 weeks post-injection of AAV-GFP or AAV-SIRT3 (upper panels). Western blot analysis for SIRT3, p16^INK4a^, and mitochondrial proteins in the SN 4 weeks and 18 months post-injection of AAV-GFP or AAV-SIRT3 (lower panels). **c** Immunohistochemical staining for TH in the SN of 3- and 20-month-old intact controls, as well as 20-month-old experimental mice 18 months post-injection with AAV-GFP or AAV-SIRT3 (scale bar, 500 μm), along with quantification of TH-positive neurons in the SNpc (upper panels). VTA, ventral tegmental area. Western blot analysis for cleaved Caspase-3 (c-Caspase-3), cleaved poly (ADP-ribose) polymerase 1 (c-PARP-1), phosphorylated-mixed lineage kinase domain-like protein (p-MLKL), and phosphorylated-Receptor-interacting serine/threonine-protein kinase 3 (p-RIP3), markers associated with cell death in the SN (middle panels). Immunohistochemical staining for TH in the striatum (STR; scale bar, 500 μm; left lower panels), and HPLC analysis for DA and HVA in the STR of mice (right lower panels). **d** Behavioral tests (rotarod and grip strength) were performed every 2 months until 20 months of age (upper panels). DXA scans for body composition and ROI analysis (middle panels). Western blotting for p16^INK4a^ and synaptophysin in muscle tissues (lower panels). Statistical analyses were performed using Student’s *t*-test, one-way or two-way ANOVA with Tukey’s post hoc test, or the Kruskal–Wallis test (for HVA and LBM). Data as mean ± SEM. *p < 0.05, **p < 0.01, ***p < 0.001 vs. 3-month-old intact or between the indicated groups (*n* = 14 for behavioral tests; *n* = 6 for other analyses)

To enhance SIRT3 expression, we administered adeno-associated virus serotype 1 (AAV1) encoding *Sirt3* into the SNpc of mice. The signals of green fluorescent protein (GFP), a marker for target expression, were highly enriched in TH-positive DA neurons 4 weeks post-AAV-SIRT3 administration (Fig. 1a, lower panels), with negligible expression in glial cells (staining data not shown). Gene expression profiling 4 weeks post-AAV-SIRT3 administration revealed that SIRT3 upregulated genes associated with mitochondrial functions, including the mitochondrial respiratory chain, ATP synthesis, and the DA signaling pathway (Fig. 1b, upper panels). Western blot analysis confirmed a significant increase in SIRT3 expression in both young and aged mice transduced with AAV-SIRT3 compared to age-matched controls, demonstrating sustained upregulation (Fig. 1b, lower panels). Additionally, SIRT3 upregulation and p16^INK4a^ expression, a senescence marker, exhibited an inversely reciprocal relationship, as shown by the reduced p16^INK4a^ expression in aged mice following SIRT3 upregulation. Moreover, mitochondria-related genes (data not shown) and proteins, including dynamin-related protein 1 (DRP1, a mediator for mitochondria fission), mitofusion 2 (MFN2, a mediator for mitochondrial fusion), ATP synthase F1 subunit alpha (ATP5A, a subunit of mitochondrial ATP synthase), cytochrome C oxidase subunit IV (COXIV, a component of mitochondrial electron transport chain), and mitochondrially encoded cytochrome *C* oxidase II (MTC02, mitochondrial non-glycosylated protein), were significantly preserved in the SN of aged mice with SIRT3 upregulation compared to untreated aged mice.

To evaluate the impact of SIRT3 upregulation on preserving nigral DA neurons during aging, we performed additional immunohistochemical staining. SIRT3 upregulation was found to preserve DA activity in aged mice, as evidenced by the sustained expression of TH-positive neurons (Fig. 1c, upper panels); this finding suggested neuroprotection over time, which was further supported by Western blot analysis (Fig. 1c, middle panels). Moreover, consistent with the patterns of striatal TH expression (Fig. 1c, lower panels), high-performance liquid chromatography (HPLC) analysis revealed a significant preservation of striatal DA and its metabolite, homovanillic acid (HVA), in aged mice with SIRT3 upregulation compared to age-matched controls (Fig. 1c, lower panels).

In line with the preservation of the nigrostriatal DA system, the age-related decline in motor function was significantly attenuated by SIRT3 upregulation in the SNpc (Fig. 1d, upper panels). Additionally, dual-energy X-ray absorptiometry (DXA) analysis revealed a reduction in lean body mass (LBM) across the whole body, including the limbs, in 20-month-old mice compared to 3-month-old mice (Fig. 1d, middle panels), while body weight and fat body mass (FBM) increased with aging (data not shown). Importantly, aged mice with SIRT3 upregulation in nigral DA neurons exhibited significantly greater muscle mass preservation compared to age-matched controls (Fig. 1d, middle panels), along with reduced FBM (data not shown). Consistent with the preservation of LBM, Western blot analysis revealed that the elevated levels of p16^INK4a^ protein observed in the skeletal muscle of 20-month-old intact mice were significantly reduced in aged mice with SIRT3 upregulation (Fig. 1d, lower panels). Furthermore, SIRT3 upregulation significantly preserved synaptophysin expression, which regulates synaptic vesicles at presynaptic nerve terminals, in the muscle tissues of aged mice compared to age-matched controls (Fig. 1d, lower panels), suggesting enhanced protection of neuromuscular junctions.

Preserving locomotor capacity is essential for improving the quality of life in the elderly. Our results demonstrate that SIRT3 upregulation via AAV-mediated *Sirt3* delivery to nigral DA neurons preserves neural activity by sustaining mitochondrial function, ultimately preventing motor decline during aging. These findings highlight the critical role of maintaining nigrostriatal DA system integrity in preventing voluntary muscle loss. Although further research is needed to elucidate the mechanisms underlying muscle mass retention and neural circuitry, a plausible hypothesis is that preserved motor function through the maintenance of the nigrostriatal DA system promotes sustained muscle mass, creating a positive feedback loop that helps maintain motor performance as aging progresses. Notably, the preservation of voluntary muscle in SIRT3-upregulated mice underscores the importance of DA neuron activity in the SN as a key strategy to counteract motor decline. Collectively, this study provides valuable insights into age-related motor deterioration and suggests novel therapeutic approaches to mitigate its effects.

## Supplementary information

Supplementary Materials

## Data Availability

The data and materials used in the current study, including all data not presented in the main text, are available from the corresponding authors upon reasonable request. The RNA-seq data used in this study can be found on NCBI GEO under accession GSE246388.
