# Supplementary material for: Enhancing the anti-aging potential of the nigrostriatal dopamine system to counteract age-related motor decline
Source: Signal Transduct Target Ther. 2025 May 12;10:153. doi: 10.1038/s41392-025-02234-7 (PMC12066703; doi:10.1038/s41392-025-02234-7)
Supplement: Supplementary file 1 — Supplementary Materials [file 41392_2025_2234_MOESM1_ESM.docx]

Supplementary Materials for

**Enhancing the anti-aging potential of the nigrostriatal dopamine system to counteract age-related motor decline**

Youngpyo Nam, Sehwan Kim, Jun-Yeong Lee, Jaekwang Kim and Sang Ryong Kim

These authors contributed equally: Youngpyo Nam, Sehwan Kim, Jun-Yeong Lee, Jaekwang Kim

Correspondence to: Sang Ryong Kim ([srk75@knu.ac.kr](mailto:srk75@knu.ac.kr))

This PDF file includes:

Methods and Materials

**Methods and Materials**

**Animals and ethics approval**

Male C57BL/6J wild-type (WT) mice (8 weeks old, ~25 g) were obtained from Hyochang Science (Daegu, Republic of Korea). The animals were housed in a temperature-controlled environment under a 12-hour light/dark cycle, with ad libitum access to food and water. They were randomly assigned to treatment groups. RNA transcriptome analysis was conducted on individual samples without pooling, with a sample size of six. All animal experiments adhered to approved protocols and guidelines, in compliance with the ARRIVE guidelines and the Animal Care Committee of Kyungpook National University (approval numbers: KNU 2019-0002, 2023-0140, and 2024-0095).

**Histological analysis**

Brain sections were washed with ice-cold 0.1 M phosphate-buffered saline (PBS) and blocked in 0.5% bovine serum albumin (BSA) in 0.1 M PBS. The sections were then incubated at 4°C for 24 hours with primary antibodies: rabbit anti-SIRT3, mouse anti-TH, rabbit anti-TH, and rabbit anti-p-TH. Afterward, the sections were rinsed with 0.5% BSA in 0.1 M PBS and incubated with the appropriate biotinylated or fluorescent secondary antibodies at room temperature (RT) for 1 hour. For DAB staining, the sections were processed at RT using avidin–biotin reagent (Vectastain ABC Kit) for 1 hour, followed by incubation in 0.5 mg/mL 3,3'-diaminobenzidine (DAB) in 0.1 M PB containing 0.003% H2O2. The stained sections were mounted on gelatin-coated slides, covered with a cover slip, and analyzed under a bright-field microscope. Fluorescence images were captured using a fluorescence microscope (Axio Imager, Carl Zeiss, Gottingen, Germany).

**Western blot analysis**

Brain and muscle tissues were homogenized in lysis buffer containing protease and phosphatase inhibitor cocktails, then centrifuged at 14,000 × g (4°C, 15 min). The supernatant was collected, and protein concentration was measured using a BCA kit. Protein samples (50 μg) were separated by SDS-PAGE, transferred to PVDF membranes, and blocked with 5% BSA in TBST. Membranes were incubated with primary antibodies at 4°C for 48 hours: rabbit anti-TH, rabbit anti-p-TH, rabbit anti-SIRT3, mouse anti-β-actin, rabbit anti-GFP, mouse anti-p16INK4a, rabbit anti-DRP1, rabbit anti-MFN2, mouse anti-ATP5A, mouse anti-COXIV, mouse anti-MTC02, rabbit anti-c-Caspase-3, rabbit anti-c-PARP-1, rabbit anti-p-MLKL, rabbit anti-p-RIP3, and mouse anti-synaptophysin. Afterward, membranes were incubated with HRP-conjugated secondary antibodies for 1 hour. Blots were developed using ECL detection reagents, and band intensity was quantified using the ImageQuant LAS 500 imager (GE Healthcare Life Sciences).

**Production and intranigral injection of AAV vectors**

The human *Sirt3* coding sequence was cloned into an AAV vector under the control of a chicken β-actin promoter to create AAV1–GFP-T2A-hSIRT3 (AAV–SIRT3) with a titer of 1 × 10¹² viral genomes/mL. GFP, used as a control, was subcloned into the same vector backbone. This AAV vector was manufactured by Vector BioLabs (Malvern, PA). For intranigral delivery, 2 μL of the vial suspension was stereotactically injected into the bilateral substantia nigra (SN) [anterior–posterior (AP): -3.5 mm, medial–lateral (ML): ±1.1 mm, dorsal–ventral (DV): -3.7 mm] of 2-month-old mice. The injection was performed using a Hamilton syringe at a rate of 0.1 μL/min, followed by a 5-minute pause to reduce reflux.

**RNA isolation and library preparation**

Total RNA was isolated from the SN of 3-month-old mice using TRIzol and purified with the miRNeasy Mini kit. RNA concentration was measured with the Quant-IT RiboGreen assay, and integrity was assessed using TapeStation. Only samples with an RNA integrity number (RIN) > 7.0 were used for library construction. A library was prepared from 1 µg of RNA per sample using the Illumina TruSeq Stranded mRNA Sample Prep kit. mRNA with poly-A tails was purified using poly-T magnetic beads, fragmented with divalent cations, and reverse transcribed into cDNA with SuperScript II and random primers. Second-strand cDNA synthesis was performed with DNA Polymerase I, RNase H, and dUTP. The cDNA was repaired, an "A" base added, and adapters ligated. The products were purified and enriched by PCR. Libraries were quantified using KAPA Library Quantification Kits for Illumina platforms and qualified with TapeStation D1000 ScreenTape. Indexed libraries were submitted for paired-end (2 × 100 bp) sequencing on an Illumina NovaSeq platform at Macrogen Inc. (Seoul, Republic of Korea).

**Sequence alignment and gene ontology (GO) assignment of differentially expressed genes (DEGs)**

Filtered raw reads were assembled and aligned to the mouse (mm10) genome using HISAT2 v2.1.0 with default options. HTSeq2 v0.12.4 was employed to calculate raw read counts. DESeq2 v1.34.0 was used to normalize the read count and determine DEGs. Genes exhibiting a fold change ≥ 2 and a false discovery rate < 0.05 were considered DEGs.

Gene product cellular localization and functional classification were analyzed using gene lists and associated GO: cellular component (CC) and GO: biological process (BP) terms, downloaded from the BioMart section of the Ensembl genome database (<http://useast.ensembl.org/biomart/martview>). Enrichment or depletion of GO terms was assessed using the hypergeometric distribution. Gene Set Enrichment Analysis (GSEA) was performed using gene lists from the aforementioned GO: BP terms.

**Behavioral tests**

Locomotor behaviors were assessed using the rota-rod, open field, and grip strength tests. Rota-rod training (10 rpm, 10 minutes per day for 3 days) was conducted prior to AAV injections. Testing was performed every two months for 20 months, with fall latency measured at an acceleration rate of 4–40 rpm. Open field tests recorded total distance traveled in a 40 × 40 × 40 cm chamber over 5 minutes, analyzed using SMART 3.0 software (Panlab, Barcelona, Spain). Grip strength was evaluated using a digital force gauge (FGP-2, NIDEC-SHIMPO Corporation, Kyoto, Japan) by lifting the mouse by its tail and allowing it to grasp the grid with its forepaws. This measurement was repeated three times, with results averaged across all tests.

**Body composition analysis and muscle tissue collection**

Body composition was evaluated using the InAlyzer peripheral dual-energy X-ray absorptiometry (DXA) system (Medikors Inc., Seongnam, Republic of Korea) with mice anesthetized with 2% isoflurane. Each mouse underwent three scans in the 75-second optimum mode, using high-energy parameters of 80 kV/1.0 mA and low-energy parameters of 55 kV/1.25 mA. The tail was manually excluded from whole-body measurements using regions of interest (ROIs). ROIs for the hindlimb were defined as the area between the knee and ankle joints, and for the forelimb, as the area from the shoulder to the wrist. Lean body mass (LBM, %) and fat body mass (FBM, %) were calculated by manually delineating the ROIs.

**Stereological and densitometric analysis in the SN**

Stereological and densitometric analyses were performed on the SN of mice. The number of TH-positive neurons in both hemispheres was quantified using the optical fractionator method with a microscope and stereological software for systematic sampling. Neurons within the counting frame were counted, and totals were estimated based on section volume and thickness, expressed as a percentage of controls. GFP-colocalized neurons and glia (astrocytes and microglia) were counted using triple immunofluorescence, with ratios expressed as a percentage of GFP-positive cells. Densitometric analysis for p-TH in the SNpc was performed by correcting for background using optical density measurements from the cerebral cortex, with results expressed relative to controls.

**Determination of DA and its metabolites in the striatum**

Striatal regions were dissected with a 2.0 mm tissue punch and immediately frozen on dry ice. Tissues were homogenized in 400 μL of 0.1 M perchloric acid with 0.1 mM EDTA, then centrifuged at 9000 rpm (4°C, 20 min). The acid-soluble fraction was filtered (0.22 µm Spin-X filter), and 10 μL of the supernatant was analyzed via HPLC-ED using a μBondapak C18 column with a mobile phase for catecholamine analysis. Striatal DA and its metabolites, HVA and DOPAC, were detected with an ESA Coulochem II detector and quantified using ChemStation software (Agilent Technologies, Santa Clara, CA).

**Statistical analysis**

Data are expressed as individual data points corresponding to each animal, with “*n*” representing the number of animals, unless otherwise specified. Graphs showing the mean and standard error of the mean (SEM) were analyzed using the GraphPad Prism 8.3.0 software (GraphPad Software, Inc., La Jolla, CA). Experimental results were evaluated a priori using the Shapiro-Wilk normality test to assess Gaussian distribution. Comparisons between two groups were performed using Student’s *t*-test, while comparisons among multiple groups were conducted using one- or two-way analysis of variance (ANOVA) followed by Tukey’s post-hoc test. The Kruskal-Wallis test was used for non-parametric analyses. Statistical significance was set at: **p* < 0.05, ***p* < 0.01, and ****p* < 0.001 versus WT mice, 3-month-old controls, or between the indicated groups.

**Availability of detailed methods**

Detailed information on the methods and materials used in this study is available from the corresponding author upon request.
